# Phenology, sterility and inheritance of two environment genic male sterile (EGMS) lines for hybrid rice

**DOI:** 10.1186/s12284-017-0169-y

**Published:** 2017-06-29

**Authors:** R. El-Namaky, P.A.J. van Oort

**Affiliations:** 1 Africa Rice Center (AfricaRice), P.B. 96, St. Louis, Senegal; 2 Rice Research & Training Center, 33717, Sakha Kafr Sheikh, Egypt; 3 Africa Rice Center (AfricaRice), 01 B.P. 2551 Bouaké, Côte d’Ivoire; 4 Crop & Weed Ecology Group, Centre for Crop Systems Analysis, Wageningen University, P.O. Box 430, 6700 AK Wageningen, The Netherlands

**Keywords:** Environment-conditioned genic male sterility (EGMS), Simulation model, Inheritance

## Abstract

**Background:**

There is still limited quantitative understanding of how environmental factors affect sterility of Environment-conditioned genic male sterility (EGMS) lines. A model was developed for this purpose and tested based on experimental data from Ndiaye (Senegal) in 2013-2015. For the two EGMS lines tested here, it was not clear if one or more recessive gene(s) were causing male sterility. This was tested by studying sterility segregation of the F2 populations.

**Results:**

Daylength (photoperiod) and minimum temperatures during the period from panicle initiation to flowering had significant effects on male sterility. Results clearly showed that only one recessive gene was involved in causing male sterility. The model was applied to determine the set of sowing dates of two different EGMS lines such that both would flower at the same time the pollen would be completely sterile. In the same time the local popular variety (Sahel 108, the male pollen donor) being sufficiently fertile to produce the hybrid seeds. The model was applied to investigate the viability of the two line breeding system in the same location with climate change (+2oC) and in two other potential locations: in M’Be in Ivory Coast and in the Nile delta in Egypt.

**Conclusions:**

Apart from giving new insights in the relation between environment and EGMS, this study shows that these insights can be used to assess safe sowing windows and assess the suitability of sterility and fertility period of different environments for a two line hybrid rice production system.

## Background

Rice hybrids often have higher yields than high-yielding inbred varieties, often between 15% and 20% higher (Virmani et al. 2003). Where local seed markets are well functioning, hybrids can play an important contribution to farmers’ livelihoods, local and regional food security. In 2010, AfricaRice initiated breeding for hybrid rice (El-Namaky and Demont 2013). To produce hybrids, a line with male sterility is crossed with a local popular variety (the male pollen donor). Resulting F_1_ seed (hybrids) benefits from the positive effects of heterosis and benefits from genes from the local popular variety, which ideally makes the F_1_ seed higher yielding yet still well adapted to the local environment. Environment-sensitive genic male sterility (EGMS), also called Photoperiod-thermo-sensitive genic male sterile (PTGMS), has been extensively used for preventing self-pollination in the production of hybrid seeds in various crops (Virmani et al. 2003, Xu et al. 2011). Compared with three-line sterile lines in a hybrid rice system, EGMS can maintain sterile line production without using restorer lines. Furthermore, a two-line hybrid rice system by application of EGMS has many advantages, including a wider range of germplasm resources used as breeding parents, higher yields, and simpler procedures for breeding and hybrid seed production (Virmani et al. 2003; Zhou et al. 2012). With the discovery of the photoperiod sensitive genic male sterility (PGMS) line Nongken 58S in rice (Shi 1985), there has been great progress in two-line hybrid rice breeding in China.

Much research has been conducted into the molecular and genetic mechanisms causing sterility (Horner and Palmer 1995; Li et al. 2007; Chen et al. 2010; Xu et al. 2011, Huang et al. 2014). Much less is known about how exactly the environment affects male sterility (Lopez and Virmani 2000; Latha et al. 2004). In a qualitative sense it is known that long daylength and high temperature can cause male sterility. But very limited research has been conducted quantifying the relationship between daylength, temperature and sterility. Such quantitative understanding is needed when advising on sowing dates. For producing hybrids, it is important to be absolutely sure that the male sterile parent is 100% sterile and to have flowering of the male sterile parent and the local popular variety at the same date. With temperature varying between years the same sowing date could in one year give 100% sterility and less than 100% in another year. By simulating with for example 20 years of weather data, we can identify those sowing dates for which regardless of weather variability, a 100% sterility can be guaranteed. Models for predicting flowering date and sterility for normal rice varieties have been developed before, for example see van Oort et al. (2011); Julia and Dingkuhn (2013); Dingkuhn et al. (2015a,b) and van Oort et al. (2015a). A key difference between these normal varieties and the EGMS lines is that in the EGMS lines a long daylength at flowering can cause sterility, while in normal varieties no such effect of daylength on sterility has been reported. To date no simulation models have been developed for hybrid rice phenology and sterility. Once a model has been calibrated and shown to be sufficiently accurate, we can use the model to answer practical questions such as:

1. Between which start and end date is the safe sowing window for producing hybrids? (i.e. 100% sterility is simulated) Taking into account weather variability within and between years
2. Will the safe sowing window change with climate change?
3. What is the safe sowing window for producing hybrids in another location?
4. Which sowing dates are best suited for multiplying (selfing) the Male Sterile Parent (sterility <50%)?
5. What combination of sowing dates will guarantee that a local popular parent line and an EGMS parent line will flower at the same date, with the EGMS line being 100% sterile and the local popular parent sufficiently fertile (sterility <50%)?

Inheritance of male sterility is important in breeding programs for locally well adapted hybrids. In the first round, an EGMS parent is crossed with a local popular variety to produce the F_1_ seeds. The F_2_ generations were obtained from self-pollination of F_1_ hybrids. From this segregated population, breeders will be able to select a subset of plants which performed best, i.e. combining the most desirable genes from the original EGMS line and the local popular variety. This subset is used as the second generation EGMS. In comparison with the first generation EGMS and F_1_ hybrids, the second generation will have more desirable genes inherited from the local popular variety as well as the genes responsible for male sterility and needed to produce hybrids. This process can be repeated, leading to hybrids ever better performing in the test environment. Clearly, for this type of breeding it is desirable that only one recessive gene causes male sterility, in which case 25% of the F_2_ population will express the EGMS trait. If two recessive genes are involved, then a much smaller fraction of the F_2_ population will have the EGMS trait (1/16 = 6.25%), thus making breeding much more cumbersome. For this reason, it is important to understand inheritance of the EGMS trait.

The first objective of this paper is to develop a model for predicting flowering date and sterility of two EGMS lines as a function of sowing date, location data and weather data. The second objective is to study the F_2_ population, to investigate how many gene(s) are involved in causing male sterility.

## Methods

We first describe our data. Next the method of studying inheritance of the EGMS trait. The third main section of the Methods describes a new phenology and sterility model and the methods used for testing and comparing models.

### Data

#### Experiments for model development

Two Environmental Genetic Male Sterile lines, IR75589-31-27-8-33 (EGMS1) and IR77271-42-5-4-36 (EGMS2) were provided by the International Rice Research Institute (IRRI). The two lines were evaluated at the Experimental Farm of the AfricaRice Sahel Regional Center, Ndiaye, Senegal (16.22 N, 16.29 W). Both lines were sown in 2013 and 2014 at a 15 days interval (two dates per month), with 3 replicates per date, to study phenology and sterility. Sowing started on 1 January 2013 and ended on 16 December 2014. The lines were well watered, well fertilised and kept free from pests and diseases. Three observations were made: flowering date, pollen sterility and spikelet sterility. The pollen sterility percentage was measured as follows. Spikelets were collected from each primary panicle and fixed in 70% ethyl alcohol. From these spikelets, 5 to 6 anthers were collected at random and smeared in iodine potassium iodide solution (1%) and examined under light microscope (40 × 10). About 200 pollen grains were examined in three different slides. All the unstained pollens were considered as sterile and the stained ones as fertile. The pollen sterility per plant was computed and expressed as percentage for each single plant and parental lines as follows:

1 $$ \mathrm{Pollen}\ \mathrm{sterility}\ \left(\%\right)=100\%\times \frac{\mathrm{No}.\mathrm{of}\ \mathrm{sterile}\ \mathrm{pollen}\ \mathrm{grains}}{\mathrm{Total}\ \mathrm{No}.\mathrm{of}\ \mathrm{pollen}\ \mathrm{grains}} $$

This dataset contained *n* = 288 data points (48 sowing dates × 3 replicates per date × 2 EGMS lines). This was the dataset used for model development. The dataset was split in two: The first half of the data (sowing dates in 2013) were used for model calibration, the second half (sowing in 2014) was used for model validation.

In 2014, an additional alternative method of measuring sterility was tried, in which sterility was calculated from the number of filled spikelets. The spikelet fertility percentage was measured as follows. Two main panicles of each plant were bagged by glassine bag just before panicle emergence to avoid out-crossing. The total of sterile and fertile spikelet was counted from the bagged panicles of all the plants in each testcross. Spikelet fertility was calculated as:

2 $$ \mathrm{Spikelet}\ \mathrm{stertility}\ \left(\%\right)=100\%\times \frac{\mathrm{No}.\mathrm{of}\ \mathrm{unfilled}\ \mathrm{spikelets}\ }{\mathrm{Total}\ \mathrm{No}.\mathrm{of}\ \mathrm{spikelets}\ } $$

Sterility calculated with the two methods Eq. (1) and Eq. (2) was statistically compared using the chi-square test.

#### Experiments for studying inheritance of EGMS sterility

In 2014, Sahel108 (popular rice variety in West Africa) was used to develop two breeding populations with both EGMS lines (Sahel108/IR75589-31-27-8-33) and (Sahel108/IR77271-42-5-4-36). The F_1_ were sown in 20 August 2014 (Wet season) and selfed to produce the F_2_ populations. In the dry season of 2015 (sowing 15 April) both parents and F_2_ populations were sown for investigating the number of genes causing EGMS sterility. For 520 F_2_ plants (Sahel108/IR75589-31-27-8-33) and 460 F_2_ plants (Sahel108/IR77271-42-5-4-36) we measured pollen fertility and spikelet fertility as described above. Next each plant was reclassified to 0 or 1, 0 (sterile) if sterility was larger than 99% and 1 (fertile) if sterility was less than 99%. A chi-square test was used to analyse the segregation pattern and determine the genes that control both EGMS. The chi-square test for fixed ratios was applied (Gomez and Gomez 1984, p. 464, Yang 1997), with the chi-square value calculated as:

3 $$ {\upchi}^2=\frac{{\left(\left|{O}_1-{E}_1\right|-0.5\right)}^2}{E_1}+\frac{{\left(\left|{O}_0-{E}_0\right|-0.5\right)}^2}{E_0} $$

Where O_0_ and E_0_ are the observed and expected number of sterile plants and O_1_ and E_1_ are the observed and expected number of fertile plants. If one gene is controlling EGMS, we would expect a 1:3 segregation, i.e. E_0_ = 1/4 x (O_0_ + O_1_) and E_1_ = 3/4 x (O_0_ + O_1_). If 2 recessive genes are causing male sterility then we expect a 1:15 segregation, i.e. E_0_ = 1/16 x (O_0_ + O_1_) and E_1_ = 15/16 x (O_0_ + O_1_).

#### Weather data/study sites

Daily weather data were taken from the AfricaRice weather stations which have over time been operational at the Ndiaye site. Weather data were used to analyse the experimental data as well as for simulating effect of weather variability on sterility over a longer period of 25 years. The weather station time series showed many gaps. Missing data were filled in with data from the POWER database (http://power.larc.nasa.gov). The POWER database is known to have systematic errors in its minimum and maximum temperature while radiation values compare well with station data (White et al. 2008). Therefore daily *T*_*min*_ and *T*_*max*_ were corrected as follows. First, bias correction parameters *b*_*0*_ and *b*_*1*_ were estimated from linear regression between available station and POWER data (Eq. 4)

4 $$ {T}_{min}\left(\mathrm{station}\right)={b}_0+{b}_1\times {T}_{min}\left(\mathrm{POWER}\right) $$

For dates where *T*_*min*_ *(station)* was available we used these values. For dates with missing station data we estimated *T*_*min*_ from POWER (Eq. 5), using *b*_*0*_ and *b*_*1*_ determined in Eq. (4).

5 $$ {T}_{min}\left(\mathrm{POWER}\mathrm{corrected}\right)={b}_0+{b}_1\times {T}_{min}\left(\mathrm{POWER}\right) $$

The same bias correction procedure was applied for the daily maximum temperature *T*_*max*_.

After model development, simulations were conducted with weather data for four environments:

- Ndiaye, Senegal, 1990–2015
- Ndiaye, Senegal, 1990–2015, with 2 °C added to daily temperatures (climate change scenario)
- Cairo, Egypt, 1983–2012
- M’Be, Ivory Coast, 1998–2015

These locations were chosen to compare simulations for sites with contrasting environments. Rice is an important crop in the Nile delta, where input levels are high and yield gaps small (van Oort et al. 2015b), where an interesting opportunity could be to raise the yield ceiling. It is of interest to know if the same EGMS lines successfully grown in Senegal can also be grown in the Nile delta, so that they could be used to develop hybrids with in Egypt popular varieties in a breeding program located in the Nile delta. Ideally we would have used weather data from a station inside the delta, instead of Cairo which is just south of it, but no weather dataset was available from inside the delta with sufficiently long time series of weather data. Therefore Cairo weather data were used. We expect this station is fairly well representative for weather in the delta.

The hybrid rice breeding program of AfricaRice is located in the Sahel station in Ndiaye in Senegal. AfricaRice has another station with facilities and expertise for breeding located in M’Be in Ivory Coast. This would be a convenient location for making crosses between the EGMS lines and locally popular varieties in Ivory Coast (rather than importing hybrids from the AfricaRice station at Senegal, because these might be less well adapted to environment and consumer preferences in Ivory Coast). We were interested in finding out if the Cairo and M’Be sites would also be suitable for hybrid production and if so, what would be the best sowing windows for hybrid production and multiplication of the EGMS lines.

### Phenology and sterility model

A model was developed for investigating which climatic variables contribute to male sterility and for predicting sterility as a function of sowing date, weather variables and genetic parameters. The model consists of three sub-models which are described in the following sections.

#### Phenology sub-model

We developed a simplified phenology model in which we considered only two phases: “SPI”, from sowing to panicle initiation and “PIFL” as the phase from panicle initiation to flowering. The panicle initiation date was not observed. We estimated the panicle initiation (PI) date as:

6 $$ {\mathrm{Date}}_{\mathrm{PI}}{=\mathrm{Date}}_{\mathrm{S}}+0.65\times \left({\mathrm{Date}}_{\mathrm{FL}}{\textstyle \hbox{-} }{\mathrm{Date}}_{\mathrm{S}}\right) $$

We simulated on a numerical scale the Development Stage (*DS*) with *DS* = 0 for sowing, *DS* = 0.65 for panicle initiation and *DS* = 1 for flowering. The number 0.65 is taken from the ORYZA2000 model (Bouman et al. 2001); previous phenological research has shown that panicle initiation occurs at around this stage. Development starts on sowing day *d*_*S*_. *DS*_*d*_ on day *d* is simulated as:

7 $$ {DS}_d=\left\{\begin{array}{cc}\hfill 0\hfill & \hfill d<{d}_S\hfill \\ {}\hfill {DS}_{d-1}+{DVR}_{S PI}\times {TI}_d\hfill & \hfill 0\le DS<0.65\hfill \\ {}\hfill {DS}_{d-1}+{DVR}_{PIFL}\times {TI}_d\hfill & \hfill 0.65\le DS<1.0\hfill \end{array}\right. $$

Were *DS*_*d*-1_ is the development stage the previous day, *DVR*_*SPI*_ and *DVR*_*PIFL*_ are model parameters estimated through model calibration and daily thermal time increment *TI*_*d*_ is a variable calculated from daily weather data. Daily *TI*_*d*_ is calculated as the average of hourly thermal time increment *TI*_*h,d*_:

8 $$ {TI}_d=\frac{1}{24}{\sum}_{h=1}^{24}{TI}_{h, d} $$

Hourly thermal time increment *TI*_*h,d*_ is calculated from hourly temperature *T*_*h*,d_, using the so-called cardinal temperatures: the base temperature *TBD* and the optimum temperature *TOD* (above which development is fastest):

9 $$ { T I}_{h, d}= \min \left(1, \max \left(0,\frac{T_{h, d}- TBD}{ T OD- TBD}\right)\right) $$

Note that according to these equation development is fastest at *T*_*h,d*_ ≥ *TOD* (leading to *TI*_*h,d*_ = 1). The assumption of no delay in development above TOD is based on van Oort et al. (2011) and Zhang et al. (2016) who showed models with no delay in development above TOD are consistently more accurate than models with slower development above TOD. Hourly temperature on day *d*, *T*_*h,d*_, was calculated from daily minimum and daily (*d*) maximum temperature *T*_*max*_ *(d)* and *T*_*min*_ *(d)* as:

10 $$ {T}_{h, d}=\frac{\left({T}_{max}(d)+{T}_{min}(d)\right)}{2}+\left({T}_{max}(d)+{T}_{min}(d)\right)\times \cos \left(0.2618\times \left( h-14\right)\right) $$

Parameters *TBD*, *TOD*, *DVR*_*SPI*_ and *DVR*_*PIFL*_ were simultaneously estimated with the pheno_opt_rice2 phenology calibration program (van Oort et al. 2011) from the experimental data. Parameter sets were calibrated separately for the two EGMS lines. Starting from the sowing day *d*_*S*_ the phenology sub-model simulates for each day *d* the development stage *DS*_*d*_ using Eqs. 7–10. The simulated panicle initiation day *d*_*PI*_ is the day *d* for which *DS*_*d*_ = 0.65 and the simulated flowering day *d*_*FL*_ is the day *d* for which *DS*_*d*_ = 1. In summary the phenology sub-model predicts the panicle initiation day *d*_*PI*_ and flowering day *d*_*FL*_ as a function of the sowing day *d*_*S*_, daily minimum and maximum temperatures *T*_*min*_ and *T*_*max*_ and parameters *TBD*, *TOD*, *DVR*_*SPI*_ and *DVR*_*PIFL*_.

#### EGMS sterility sub-model

Three types of genic male sterility exist (Virmani et al. 2003, Xu et al. 2011): PGMS lines are completely or highly sterile under long day lengths, TGMS lines are completely or highly sterile under high or low temperatures and in photothermosensitive genetic male sterile (PTGMS) lines sterility is determined by both daylength (=photoperiod) and temperature. The more general term EGMS lines (E stands for environment) is used to represent all types. Sterility was measured on a numerical scale from 0 to 1 (=0-100%). From the experimental data we fitted logistic regression models to predict sterility *S*_*EGMS*_ from different logit functions *g(X)*, where *X* refers to different sets of explanatory variables:

11 $$ {S}_{EGMS}=\frac{e^{g(X)}}{1+{e}^{g(X)}} $$

Available research suggests that *S*_*EGMS*_ is most strongly affected by environmental conditions in the phase from panicle initiation to the 50% flowering date (Lopez and Virmani 2000; Latha et al. 2004), also in other crops with EGMS (Yuan et al. 2008). We therefore calculated average values over the period from panicle initiation to flowering (PIFL) for the following explanatory variables: *T*_*min*_(*PIFL*), *T*_*avg*_(*PIFL*), *T*_*max*_(*PIFL*) and *DAYL*_*−6*_(*PIFL*), where *T*_*min*_, *T*_*max*_ and *T*_*avg*_ are daily minimum, daily maximum and daily average temperature and *DAYL*_*−6*_ is the daily daylength including civil twilight (sun angle 6° below horizon). We also tested for differences between the two EGMS lines (recoded to 0 for EGMS1 and 1 for EGMS2). The logit function with minimum temperature *T*_*min*_ *(PIFL),* daylength and EGMS line as explanatory variables is defined as:

12 $$ g={b}_{E0}+{b}_{E1}\times {DAYL}_{-6}(PIFL)+{b}_{E2}\times {T}_{min}(PIFL)+{b}_{E3}\times EGMS $$

For all models we tested using the standard t-test if parameters *b*_*E*1_, *b*_*E*2_ and *b*_*E3*_ differed significantly from zero. Daylength *DAYL*_*sa*_ *(d)* on day *d* with sun angle *sa* was calculated using equations presented in Goudriaan and van Laar (1994). The sun angle *sa* is used for twilight: 0° means no twilight, −6° is civil twilight, i.e. including the time before sunrise and after sunset when the sun is less than 6 degrees below the horizon. Daylength at latitude *LAT* on day *d* was calculated as:

13 $$ a= \sin \left( LAT\times \frac{\pi}{180}\right)\times \sin \left(\delta \right) $$

14 $$ b= \cos \left( LAT\times \frac{\pi}{180}\right)\times \cos \left(\delta \right) $$

15 $$ \delta (d)=-\mathrm{asin}\left( \sin \left(23.45\ \frac{\pi}{180}\right)\times \cos \left(2\ \pi \frac{\left( d+10\right)}{365}\right)\right) $$

16 $$ {DAYL}_{sa}(d)=12\left(1+\frac{2}{\pi}\times \mathrm{asin}\left(\left(- \sin \left( sa\times \frac{\pi}{180}\right)+ a\right)/ b\right)\right) $$

**Fig. 1:**
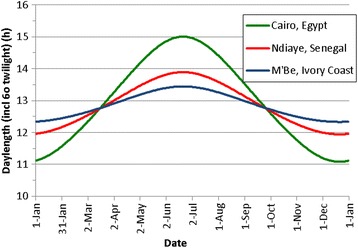
Daylength for the three study locations. Experiments were done in Ndiaye in Senegal, simulations were done for all locations

We simulated sterility for three different locations at different latitudes. Figure 1 shows daylength for Ndiaye Senegal, M’Be in Ivory Coast and Cairo in Egypt.

#### Cold sterility sub-model

When the varieties were flowering in December, January and February the EGMS sterility sub-models predicted zero sterility while observed sterility was 10-50%. We hypothesised this would be due to cold induced sterility, a phenomenon that has been documented before for the same study site for other varieties flowering in these months (Dingkuhn et al. 2015a, van Oort et al. 2015a). From these previous studies we know cold induced sterility is most strongly correlated with minimum temperature in the period from panicle initiation to flowering. Water temperature (which was not measured) can during part of this phase be a better predictor than air temperature because initially the panicle meristem is a ground height. With lack of data on water temperature we used air temperature. For parameter estimation we used the subset of data with flowering in the period of December to February, to avoid confounding effects of the EGMS type of sterility discussed in the previous section. Also here, we considered three candidate explanatory variables:

- *T*_*min*_(*PIFL*) = Average of daily minimum temperatures, averaged over the period from panicle initiation to flowering
- *T*_*avg*_(*PIFL*) = Average of daily average temperatures, averaged over the period from panicle initiation to flowering
- *T*_*max*_(*PIFL*) = Average of daily maximum temperatures, averaged over the period from panicle initiation to flowering

For each, a logistic regression model was fitted. For example for *T*_*min*_ *(PIFL)*:

17 $$ {S}_{C old}=\frac{e^{b_{C0}+{b}_{C1}\times {T}_{min}(PIFL)}}{1+{e}^{b_{C0}+{b}_{C1}\times {T}_{min}(PIFL)}} $$

#### Full model

The full model is a combination of the above three sub-models:

1. Phenology sub-model: predict panicle initiation date *d*_*PI*_ and flowering date *d*_*FL*_
2. EGMS sterility sub-model: predict photoperiod and/or temperature induced sterility *S*_*EGMS*_
3. Cold sterility sub-model: predict cold induced sterility *S*_*Cold*_
4. Total sterility *S*_*Total*_ as the maximum of the two sterilities:

18 $$ {S}_{Total}= \max \left({S}_{EGMS},{S}_{Cold}\right) $$

Table 1 lists the input and output variables and the model parameters. The final model selected consisted of 9 parameters for each EGMS line and was used to predict PI date, Flowering date, *S*_*Cold*_, *S*_*EGMS*_ and *S*_*Total*_ for *n* = 144 observations per EGMS line (48 sowing dates × 3 replicates). Of these 9 parameters 8 parameters turned out to be identical for the two EGMS lines. In total therefore, 10 parameters were used to predict phenology and sterility of *n* = 288 observations. Half of the observations was used for calibration, half for validation, the procedure for calibration/validation and accuracy assessment is discussed in the following section.

**Table 1:** Input variables, output variables and parameters

Name	Type^a^	Description	Sub-model	Table/Figure
*T*_*min*_	input	Daily minimum temperature (°C)	Phenology, EGMS Sterility, Cold Sterility	Fig. 3
*T*_*max*_	input	Daily maximum temperature (°C)	Phenology, EGMS Sterility, Cold Sterility	Fig. 3
*T*_*min*_(*PIFL*)	input	*T*_*min*_, averaged over period from panicle initiation (PI) to flowering (FL)	Phenology, EGMS Sterility, Cold Sterility	
*T*_*max*_(*PIFL*)	input	*T*_*max*_, averaged over period from panicle initiation (PI) to flowering (FL)	Phenology, EGMS Sterility, Cold Sterility	
*DAYL*_*sa*_ *(d)*	input	Daylength (h) on day *d*, calculated with sun angle *sa* (^o^) to account for twilight	EGMS Sterility	Fig. 1
*DAYL*_*−6*_ *(PIFL)*	input	Daylength (h) including civil twilight (*sa* = −6°), averaged over the period from panicle initiation (PI) to flowering (FL)	EGMS Sterility	
*LAT*	input	Latitude (decimal degrees)	Phenology, EGMS Sterility	
*d*_*S*_	input	Sowing day of year (Julian day, 1 to 365)	Phenology	Figs. 2 and 4
*d*_*PI*_	output	Panicle initiation day	Phenology, EGMS Sterility, Cold Sterility	
*d*_*FL*_	output	Flowering day	Phenology, EGMS Sterility, Cold Sterility	Fig. 4
*S*_*EGMS*_	output	Sterility due to photoperiod and high temperature (Eqs. 11 and 12)	EGMS sterility	Figs. 2 and 5
*S*_*cold*_	output	Sterility due to cold (Eq. 17)	Cold Sterility	Figs. 2 and 6
*S*_*Total*_	output	Total sterility (Eq. 18)	Full model	Fig. 2
*TBD*	param.	Base temperature for development (°C)	Phenology	Table 4
*TOD*	param.	Optimum temperature for development (°C)	Phenology	Table 4
*DVR*_*SPI*_	param.	Development rate for the phase from sowing (DS = 0) to panicle initiation DS = 0.65 (d^−1^)	Phenology	Table 4
*DVR*_*PIFL*_	param.	Development rate for the phase panicle initiation DS = 0.65 (d^−1^) to flowering (DS = 1)	Phenology	Table 4
*b*_*E0*_	param.	EGMS Sterility parameter (Eqs. 11 and 12)	EGMS Sterility	Table 5
*b*_*E1*_	param.	EGMS Sterility parameter (Eqs. 11 and 12)	EGMS Sterility	Table 5
*b*_*E2*_	param.	EGMS Sterility parameter (Eqs. 11 and 12)	EGMS Sterility	Table 5
*b*_*C0*_	param.	Cold Sterility parameter (Eq. 17)	Cold Sterility	Table 6
*b*_*C1*_	param.	Cold Sterility parameter (Eq. 17)	Cold Sterility	Table 6

^a^input variable, output variable, parameter

#### Model accuracy

The experimental data set used for model calibration and validations consisted of 24 sowing dates in 2013 and 24 sowing dates in 2014, with 2 EGMS lines and 3 replicates per sowing date. The data for the sowing dates in 2013 were used for calibration and the data for sowing dates in 2014 for validation. Accuracy in simulated days from sowing to flowering and simulated sterility was measured as root mean square error (RMSE) and as modelling efficiency (EF) (Nash and Sutcliffe, 1970):

19 $$ RMSE=\sqrt{\frac{\sum {\left({S}_i-{O}_i\right)}^2}{n}} $$

20 $$ EF=1-\frac{\sum {\left({S}_i-{O}_i\right)}^2}{\sum {\left(\overline{O}-{O}_i\right)}^2} $$

Where *S*_*i*_ is the simulated variable (sterility or days from sowing to flowering) in treatment *i*, *O*_*i*_ is the observed variable in treatment *i,* $\overline{O}$ is the average of observed variables and *n* the number of observations. A value of 1 for EF indicates perfect prediction. For comparison of the logistic regression models we also used the Akaike Information Criterion (AIC), a standard measure for the goodness of fit of logistic regression models. A lower AIC value indicates a more accurate model.

#### Model applications

We used the full model to answer the following questions:

1. In Ndiaye, Senegal, which sowing give a simulated male sterility *S*_*EGMS*_ = 1 = 100%? (period for producing hybrids by crossing with local popular varieties)
2. In Ndiaye, Senegal, which sowing dates give a simulated sterility *S*_*Total*_ < 50%? (period for multiplication of the EGMS lines through self-pollination)
3. In Ndiaye, Senegal, will simulated sterility change with 2 °C temperature increase?
4. Cairo, Egypt: can the same EGMS lines and hybrids also be produced there?
5. M’Be, Ivory Coast: can the same EGMS lines and hybrids also be produced there?

## Results

### Fertility behaviour of EGMS lines with daylength and temperature

**Fig. 2:**
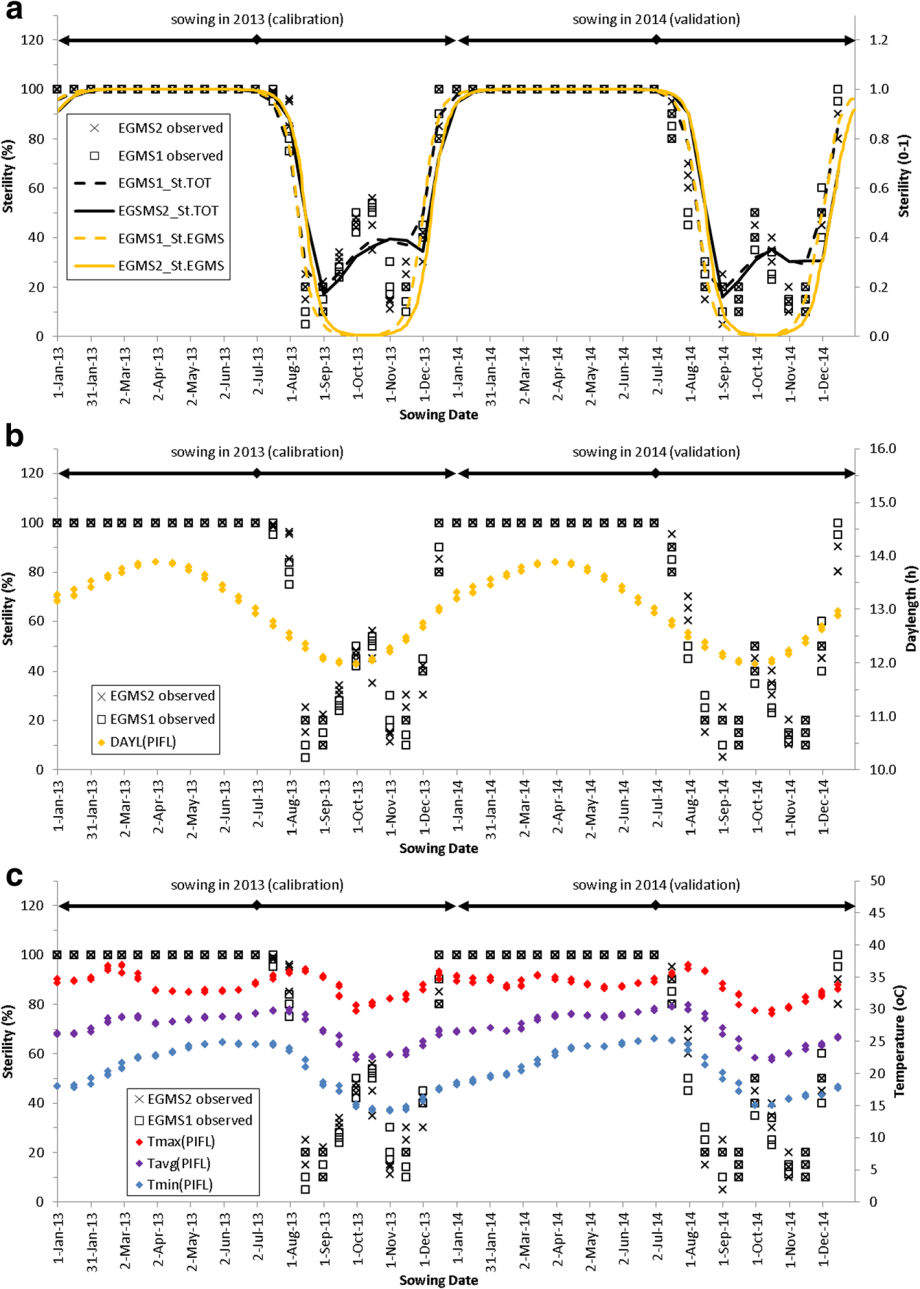
Fertility behaviour of EGMS lines with daylength and temperature. *Black symbols* show observed sterilities for the two EGMS lines. In **a** *black lines* show the simulations with the full model, *yellow lines* show simulated sterility with the EGMS model (without cold sterility). In **b**, the right axis and *yellow symbols* show the average daylength in the period from panicle initiation to flowering (PIFL) for each sowing date. In **c** the right axis shows air temperature and *coloured symbols* show the average of daily maximum, daily average and daily minimum temperatures, averaged over the PIFL period associated with each sowing date

In Fig. 2, we show the observed sterility at 48 sowing dates covering two years. In the top pane (Fig. 2a) we show in lines the simulated sterility for the full model. In the middle pane (Fig. 2b) we show average daylength in the simulated period from panicle initiation to flowering (PIFL). In the bottom pane (Fig. 2c) we show temperatures in the simulated period from panicle initiation to flowering. In the (Appendix 1: Fig. 9), we present the same figure but with flowering date instead of sowing date on the x-axis. In terms of sterility, four distinct periods can be identified from the Figure:

1. Sowing dates from 1 January to mid-July: Constant high sterility *S*_*EGMS*_ = 100%. This is a suitable period for sowing to produce hybrids.
2. Sowing dates from mid-July to 1 September: Decreasing sterility. *S*_*EGMS*_ is declining because of shortening daylengths and temperatures in the September-November period (the panicle initiation to flowering period associated with these sowing dates).
3. Sowing dates from 1 September to mid-November: Moderate to low sterility. *S*_*Cold*_ is 10-50% due to cold, *S*_*EGMS*_ (yellow lines) is around 0%.
4. Sowing dates from mid-November to 1 January: Increasing sterility. *S*_*EGMS*_ is increasing because of increasing daylengths and temperatures in the February-April period (the panicle initiation to flowering period associated with these sowing dates).

The full model (Fig. 2a) predicted sterility with RMSE values of 8 to 12% and model efficiencies of 0.88 to 0.94 (Table 2). Model accuracies were consistently a bit higher for EGMS1 than for EGMS2 and consistently a bit higher for the calibration than for validation. For cold sterility our model underestimated cold sterility for the October sowing dates and overestimated cold sterility in the November sowing dates. Those crops sown in November are right from the start of the growing period exposed to colder temperatures. This suggests that possibly some process of acclimation is occurring (as has also been hypothesised by Dingkuhn et al. 2015a). The sparsity of our data does not allow for further investigating this hypothesis. Given this period of cold we can identify two periods best suited for multiplication of the EGMS lines, i.e. where sterility is lowest. These are (1) sowing in mid august to mid September and (2) sowing in November. Associated flowering dates are November and March, just outside the coldest part of the year, the December-February period.

**Table 2:** Accuracy of total sterility simulations

		EGMS1	EGMS2
RMSE^a^	Calibration	8.2%	10.4%
	Validation	9.3%	12.0%
EF	Calibration	0.94	0.90
	Validation	0.93	0.88

^**a**^RMSE = Root mean square error (Eq. 19)

*EF* = Modelling efficiency (Eq. 20)

From Fig. 2 we can already roughly determine daylength and temperature thresholds. The period of complete sterility starts at early January. The associated flowering date is early May. The associated daylength (incl twilight) in the PIFL period is 13.3 h (Fig. 2b) and average temperature (T_avg_) in the PIFL period for this sowing date is 26 °C. The period with complete sterility ends with sowing early July (flowering early October), with daylength *DAYL*_*−6*_ *(PIFL)* = 12.9 h and *T*_*avg*_ *(PIFL)* is 30 °C. Here temperature is higher and daylength is shorter than for the January sowing date. This suggests that both temperature and daylength play a role, that at higher temperatures the critical daylength will be shorter. We investigate this hypothesis in more detail in the following sections.

### Inheritance of sterility

The F_1_ hybrids of both populations of IR75589-31-27-8-33/Sahel108 (EGMS1/Sahel108) and IR77271-42-5-4-36/Sahel108 (EGMS2/Sahel108) sown in 20 August 2014 were completely fertile. Sterilities in the F_2_ population sown in 15 April 2015 clearly indicated that only one recessive gene is involved in causing male sterility (Table 3). For IR77271-42-5-4-36/Sahel108 we found 23% pollen sterility and 27% spikelet sterility (104/460 and 125/460). For IR75589-31-27-8-33/Sahel108 we found 27% pollen sterility and 23% spikelet sterility. These fractions did not differ significantly from the expected sterility of 25% (*p*-value ranging from 0.21 to 0.31). Observed sterilities did differ significantly from the sterility of 6.25% which we would expect if two recessive genes were involved. For both F_2_ populations, the difference between pollen sterility and spikelet sterility was statistically significant, but in absolute terms the difference was small (+4% and −4%).

**Table 3:** Pollen and spikelet fertility of F_2_ populations

	Total number of plants	Observed		Expected		*χ* ^*2*^ (*p*-value)
		Fertile	Sterile	Fertile (75%)	Sterile (25%)	
Pollen fertility						
IR75589-31-27-8-33/Sahel 108	520	378	142	390	130	1.36 (0.24)
IR77271-42-5-4-36/Sahel 108	460	356	104	345	115	1.28 (0.26)
Spikelet fertility						
IR75589-31-27-8-33/Sahel 108	520	403	117	390	130	1.60 (0.21)
IR77271-42-5-4-36/Sahel 108	460	335	125	345	115	1.05 (0.31)

### Phenology and sterility model

#### Phenology

**Fig. 3:**
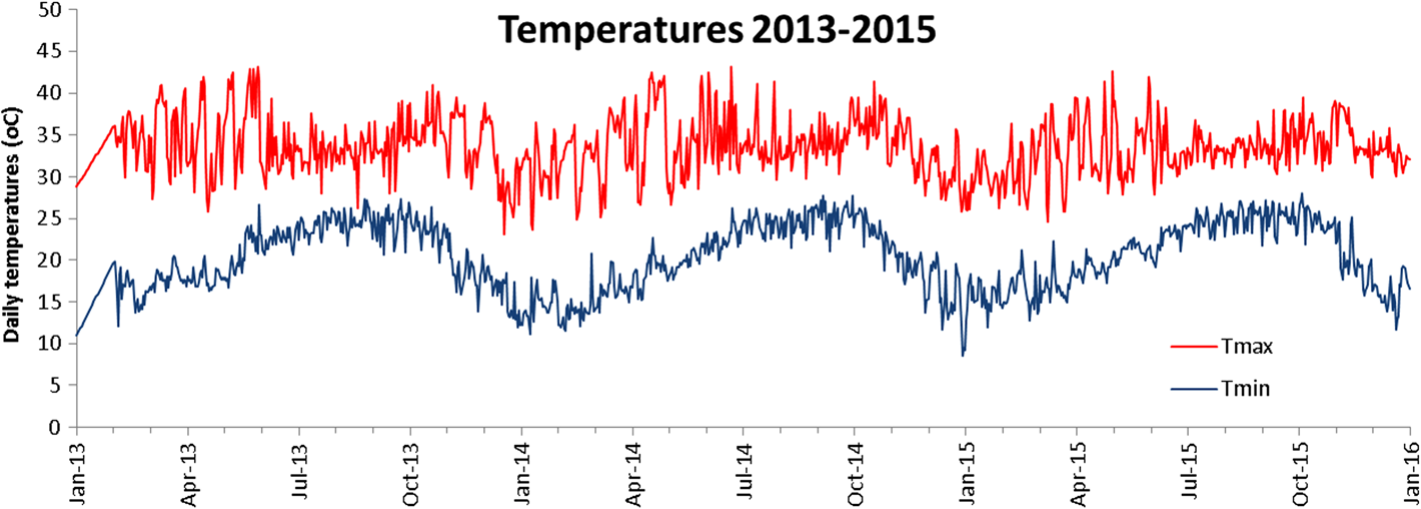
Daily minimum and maximum air temperatures during the experimentation period

**Fig. 4:**
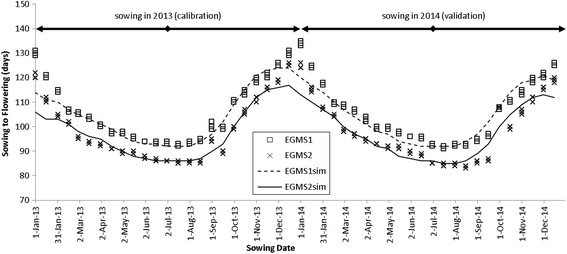
Days from sowing to flowering. Lines show simulated sterility, points show observed data. Observed data for sowing dates in 2013 were used for model calibration, data for sowing dates in 2014 were used for validation. The model predicts very accurately for the February to November period and less accurately for sowing dates in December and January

Figure 3 shows temperatures over the three years used for calibration and validation. Lower temperatures in December to February lead to longer duration from sowing to flowering when sown in November to January. Days from sowing to flowering differed significantly between the two EGMS lines, with EMGS1 having 7–14 days longer duration. A phenology model with a base temperature of 11 °C, an optimum temperature of 26 °C and no delay in development above the optimum temperature accurately predicted the days to flowering (Table 4, Fig. 4). RMSE was 4 days and model efficiency EF was 0.89, both for the calibration and the validation and for both of the two EGMS lines (Table 3). Such accuracies are normal for phenology simulations (van Oort et al. 2011, Dingkuhn et al. 2015b, Zhang et al. 2016). A visual analysis revealed that for sowing in mid December to mid January, the model systematically predicted a too short duration, with maximum errors of 15 days.

**Table 4:** Phenology parameters and model accuracy

Parameter	EGMS1	EGMS2	Description
TBD (°C)	11	11	Base temperature for development
TOD (°C)	26	26	Optimum temperature for development^a^
DVR_SPI_ (d^−1^)	0.011818	0.013265	Development rate for the phase from sowing (DS = 0) to panicle initiation DS = 0.65
DVR_PIFL_ (d^−1^)	0.010000	0.010294	Development rate for the phase panicle initiation (DS = 0.65) to flowering (DS = 1)
Accuracy	EGMS1	EGMS2	
RMSE_SFL_ (d)	4.0	4.2	Calibration Accuracy: Root mean square error for the duration from sowing to flowering
RMSE_SFL_ (d)	4.0	4.2	Validation accuracy
EF	0.89	0.89	Calibration Accuracy: Model Efficiency for the duration from sowing to flowering
EF	0.89	0.88	Validation accuracy

^a^We assumed that above TOD, development rate remains optimal (Eq. 9)

#### EGMS sterility sub-model

**Fig. 5:**
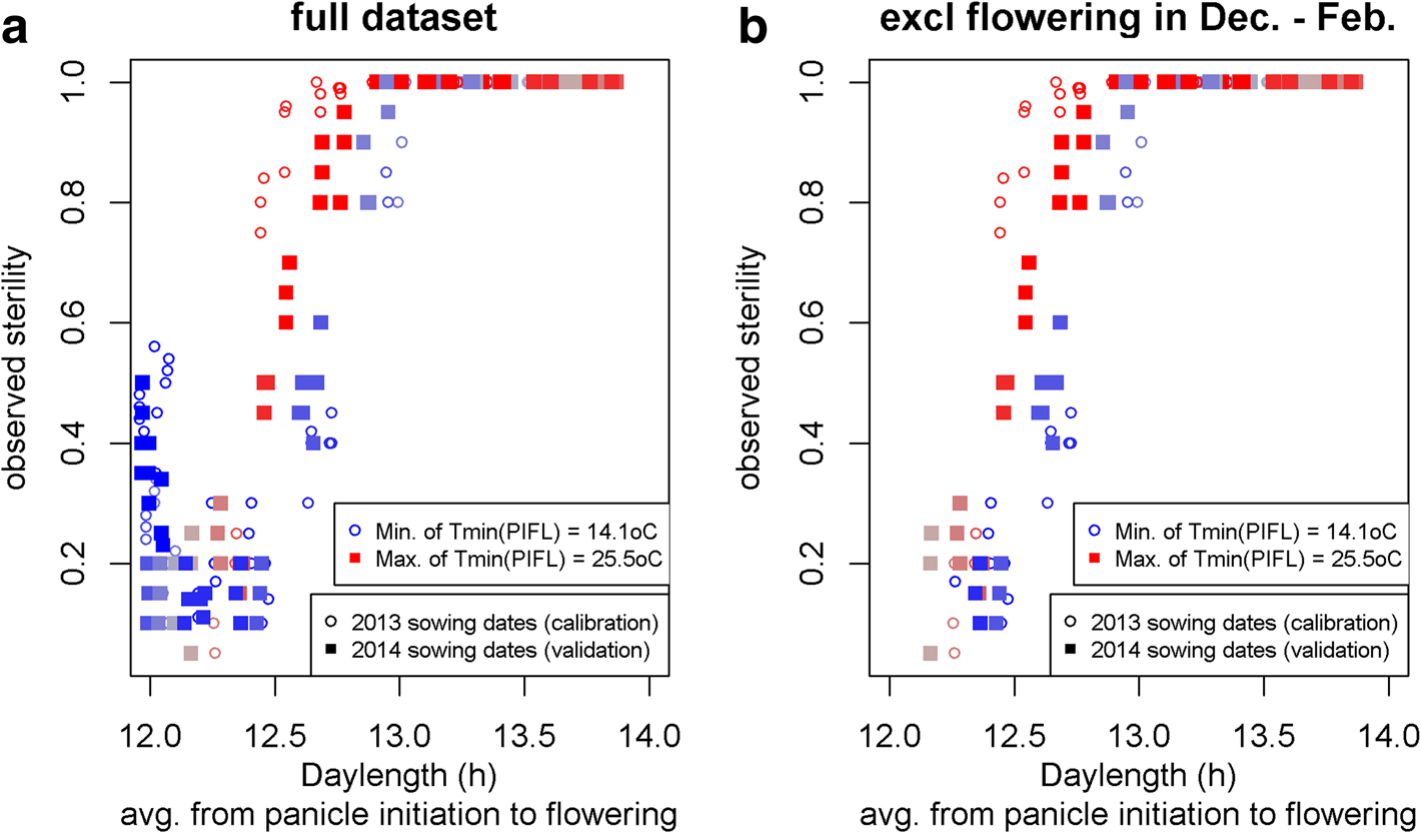
Relation between observed sterility and daylength. The colours from *blue* to *red* show the average of daily minimum temperatures (T_min_) averaged over the period from panicle initiation to flowering (PIFL). In **a** the full dataset is shown, with a spike in observed sterilities at short daylengths (12.0 hours) which is probably caused by cold sterility. In **b** the dataset is shown excluding data points with flowering dates in December to February. The dataset in **b** was used for calibration (*open circles*) and validation (*solid squares*) of the EGMS model

Figure 5 shows the relation between daylength (incl civil twilight) from panicle initiation to flowering. Figure 5a shows the full dataset, including the 10-50% sterilities for flowering in December to February (when days are short) which are caused by cold sterility. To avoid confounding effects, these were not used for model calibration. For calibration we used the open symbols (sowing in 2013) from data shown in Fig. 5b. In Fig. 5b we can clearly see a sigmoid shape of sterility increasing with daylength, but we can also see from the colours that in the transition zone from 12.5 to 13.0 h daylength, sterility is higher when *T*_*min*_(*PIFL*) is higher.

Table 5 shows the fitted models with asterices (*) for significance for the parameters. Both *T*_*min*_(*PIFL*) and *DAYL*_*−6*_(*PIFL*) had statistically significant effects. Model accuracy of the *DAYL*_*−6*_(*PIFL*) model was much higher than that of the *T*_*min*_(*PIFL*) based model (compare models 1 and 2: AIC = 43 and 73). *T*_*min*_(*PIFL*) and *DAYL*_*−6*_(*PIFL*) were only weakly correlated (R^2^ = 0.11) which means their effects can be separately investigated. In the model that incorporated both these variables (model 6, AIC = 25), both showed a significant effect, with a lower *p*-value for *DAYL*_*−6*_(*PIFL*) than for *T*_*min*_(*PIFL*). Next we investigated if models with interaction terms (models 10 and 15) would predict more accurately than the additive model. This was not the case. The model with only the interaction term *T*_*min*_(*PIFL*) x *DAYL*_*−6*_(*PIFL*) had a higher AIC value than the best model without interaction (compare models 1 and 15, AIC = 43 and 61), thus model 1 is more accurate. In the model with both additive and interaction effects (model 10), none of the parameters differed significantly from zero and the AIC value (AIC = 24) is almost the same as that of model 6 (AIC = 25). We therefore consider the additive model 6 the most accurate, with *T*_*min*_(*PIFL*) and *DAYL*_*−6*_(*PIFL*) as the explanatory variables.

**Table 5:** EGMS models calibrated from the 2013 sowing dates

Model	Equation^1^	AIC^2^
1	−84.272*** + 6.737*** x *DAYL*_*−6*_(*PIFL*)	43
2	−6.083** + 0.400*** x *T*_*min*_(*PIFL*)	73
3	−14.571*** + 0.606*** x *T*_*avg*_(*PIFL*)	76
4	−3.545 + 0.158 x *T*_*max*_(*PIFL*)	106
5	1.940*** + 0.215x*EGMS*	108
6	−95.586*** + 7.120*** x *DAYL*_*−6*_(*PIFL*) + 0.330* x *T*_*min*_(*PIFL*)	25
7	−100.627*** + 7.075*** x *DAYL*_*−6*_(*PIFL*) + 0.446* x *T*_*avg*_(*PIFL*)	27
8	−108.282*** + 7.291*** x *DAYL*_*−6*_(*PIFL*) + 0.495 x *T*_*max*_(*PIFL*)	36
9	−84.328*** + 6.730*** x *DAYL*_*−6*_(*PIFL*) + 0.092 x *EGMS*	46
10	316.130 - 25.809 x *DAYL*_*−6*_(*PIFL*) - 22.142 x *T*_*min*_(*PIFL*) + 1.799 x *DAYL*_*−6*_(*PIFL*) x *T*_*min*_(*PIFL*)	24
11	760.139 - 61.840 x *DAYL*_*−6*_(*PIFL*) - 31.919 x *T*_*avg*_(*PIFL*) + 2.592 x *DAYL*_*−6*_(*PIFL*) x *T*_*avg*_(*PIFL*)	24
12	430.004 - 35.931 x *DAYL*_*−6*_(*PIFL*) - 15.359 x *T*_*max*_(*PIFL*) + 1.273 x *DAYL*_*−6*_(*PIFL*) x *T*_*max*_(*PIFL*)	36
13	−64.587 + 5.160 x *DAYL*_*−6*_(*PIFL*) - 13.867 x *EGMS* + 1.111 x *DAYL*_*−6*_(*PIFL*) x *EGMS*	47
14	−95.704*** + 7.112*** x *DAYL*_*−6*_(*PIFL*) + 0.330* x *T*_*min*_(*PIFL*) + 0.142 x *EGMS*	27
15	−7.163*** + 0.035*** x *DAYL*_*−6*_(*PIFL*) x *T*_*min*_(*PIFL*)	61

*DAYL*_*−6*_(*PIFL*) is the average of daylengths from panicle initiation to flowering, calculated with civil twilight (sun up to 6° below the horizon). *T*_*min*_(*PIFL*) is the average of daily minimum temperatures *T*_*min*_ from panicle initiation to flowering. EGMS is a binary: 0 for EGMS1, 1 for EGMS2

^1^Significance codes: * *p* < 0.05, ** *p* < 0.01, *** *p* < 0.001

^2^Akaike Information Criterion (lower is better)

No statistically significant effects of *T*_*max*_(*PIFL*) and *EGMS* on sterility were found. Significant effects of *T*_*avg*_(*PIFL*) were found. But considering that *T*_*avg*_ is calculated as the average of *T*_*min*_ and *T*_*max*_ and given that the effect of *T*_*max*_ was not significant we think the significant effects of *T*_*avg*_ found here are the result of strong correlation between *T*_*min*_ and *T*_*avg*_ (R^2^ = 0.84) and not an effect of *T*_*avg*_ per-se.

#### Cold sterility sub-model

**Fig. 6:**
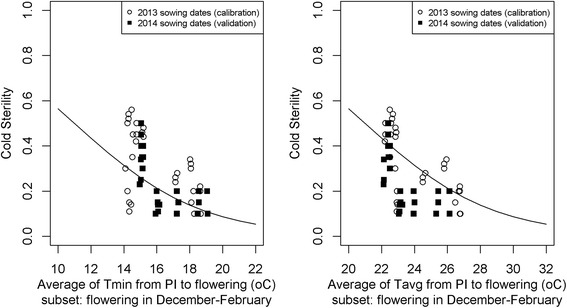
Relation between cold sterility and temperature. Data points are observed values for 2 EGMS lines, sown at 24 dates (15 day interval) from 2012 to 2013, with 3 replicates per EGMS line and sowing date, for the subset of data points with flowering dates in December to February. *Lines* are regression lines based on parameters in Table 5

Figure 6 shows two calibrated cold sterility sub-models, with parameters listed in Table 6. As with the EGMS sterility model, no significant differences were found between the EGMS lines in terms of cold sterility (result not shown). Although the regression line in Fig. 6 showed increasing sterility at lower temperatures, statistical testing showed the regression parameter *b*_*C1*_ did not differ significantly from zero (*p* > 0.1). Possibly these high *p*-values are caused by lack of power (only *n* = 56 data points had flowering in December-February, of which *n* = 29 were sown in 2013 and used for calibration). Outside the December-February period temperatures were higher, but sterility was not lower (as we would expect from Fig. 6) because for flowering dates outside the December-February period sterilities increase again due to EGMS sterility. As a result of this, we could not find lower *p*-values by expanding the dataset with data from more sowing dates. Aside from this lack of power, low accuracies may be caused by uncertainties in the model. In particular, three uncertainties can be identified. Firstly, we know theoretically that water temperature is a better predictor than air temperature, but water temperature data were not available and therefore we used air temperature as a proxy. A second uncertainty is that we used estimated and not observed panicle initiation dates and this introduces some uncertainty in the estimation of temperature in the period from panicle initiation to flowering. A third uncertainty here is that the temporal window of the cold sensitive period was potentially wrong. We discuss this in more detail in the discussion section of this paper.

**Table 6:** Cold Sterility models calibrated from the 2013 sowing dates

Model	Equation^1^	AIC^2^
1	2.5095 - 0.2056 x *T*_*min*_(*PIFL*)	29
2	5.4650 - 0.2604 x *T*_*avg*_(*PIFL*)	29
3	8.1892 - 0.2804 x *T*_*max*_(*PIFL*)	30

*T*_*min*_(*PIFL*) is the average of daily minimum temperatures *T*_*min*_ from panicle initiation to flowering. *T*_*avg*_(*PIFL*)is the average of daily average temperatures and *T*_*max*_(*PIFL*)is the average of daily maximum temperatures over this period

^1^Significance codes: * *p* < 0.05, ** *p* < 0.01, *** *p* < 0.001

^2^Akaike Information Criterion (lower is better)

The results however confirm previous research on cold sterility effects and are our only plausible explanation for the spike in sterility for flowering dates in December – February, which cannot be explained from EGMS sterility (Figs. 2a, 5a). We therefore included the cold sterility sub-model in the full model despite the non-significant *b*_*C1*_ parameter. We included the model with *T*_*min*_(*PIFL*) because previous research for non-EGMS varieties (Dingkuhn et al. 2015a, van Oort et al. 2015a) showed that *T*_*min*_ is a better predictor for cold sterility than *T*_*avg*_ or *T*_*max*_.

#### Full model

For the full model, we used the three sub-models discussed in the above sub-sections. For EGMS sterility, we used EGMS model 6 (Table 5). For cold sterility we used Cold sterility model 1 (Table 6). Overall accuracy was already presented above.

### Model applications

We present two applications of the model: site selection and synchronisation of flowering dates of two the male and female parents of F_1_ seed.

#### Suitability of different sites

**Fig. 7:**
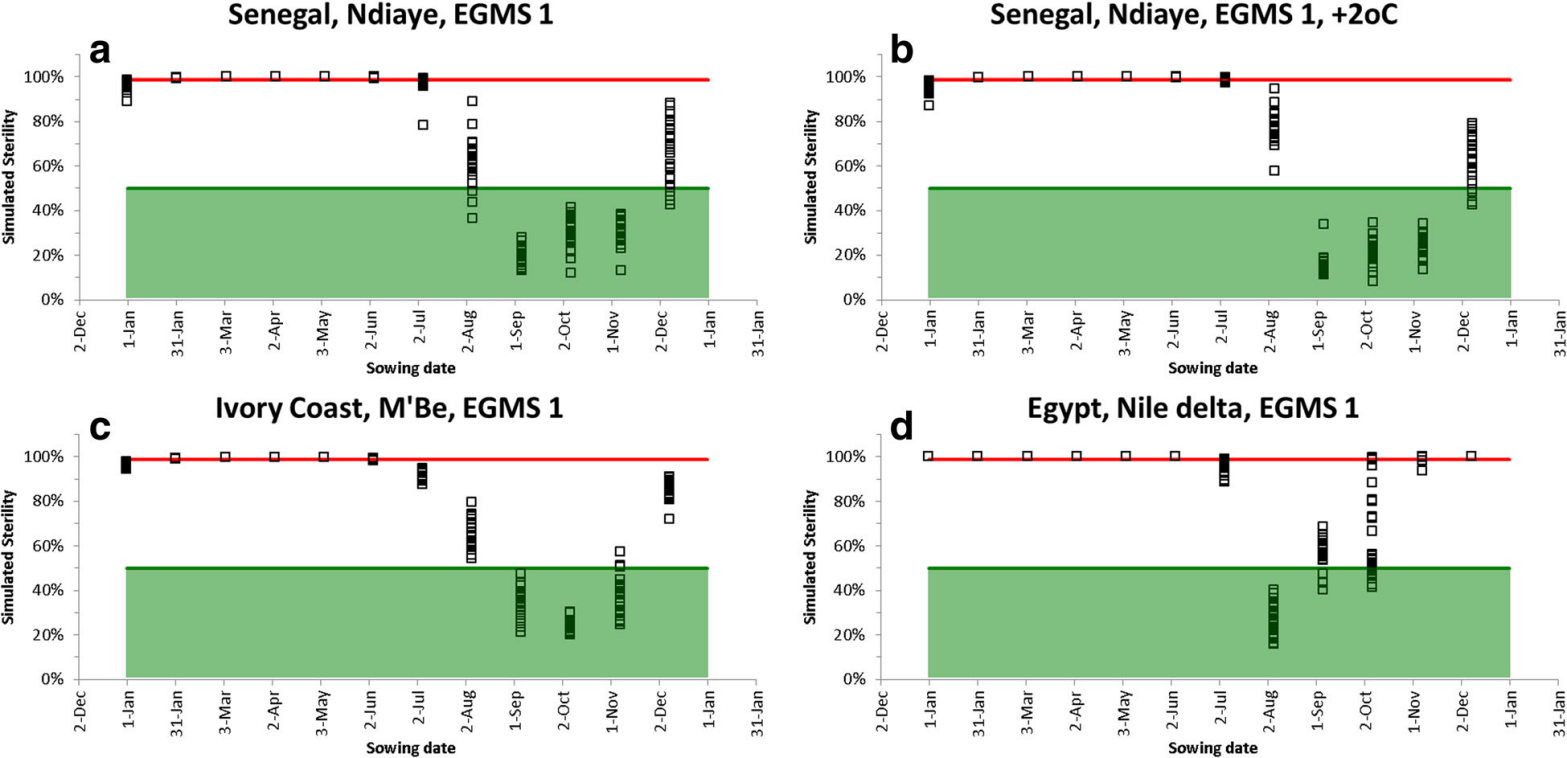
Simulated total sterility at monthly sowing dates for 4 environments. **a** Ndiaye Senegal, **b** Ndiaye Senegal with 2oC temperature increase, **c** M’Be in AfricaRice and **d** Nile Delta in Egypt. Points at or above the red line are suitable for production of F_1_ (hybrid) seed because the EGMS parent line is completely sterile. Points in the green area show suitable dates for multiplication (through self-pollination) of the EGMS line

We used the full model to identify two distinct periods for production of hybrids, namely a period with 100% sterility and a period with <50% sterility. Fig. 7 shows simulated sterility for monthly sowing dates for different environments. Points above the red line are suitable sowing dates for producing hybrids (*S*_*EGMS*_ = 100%). Points in the shaded green area (*S*_*Total*_ < 50%) are suitable sowing dates for multiplication of the EGMS line. We show here only simulation results for EGMS1 as previous analyses (Fig. 2) revealed only small differences between the two EGMS lines. We can see from Fig. 7a, b that climate change changes sterilities a bit, but changes are too small to be consequential. With +2 °C climate change suitable sowing dates for hybrid production and EGMS multiplication (cross-pollination for producing F_1_ seed and self-pollination to maintain the EGMS line) remain the same as in the current climate. Ndiaye in Senegal has a period of 6–7 sowing months suitable for production of hybrids and a 3–4 months period suitable for multiplication of the EGMS line. The AfricaRice station in M’be in Ivory coast has a period of 5–6 sowing months suitable for production of hybrids and a 3–4 months period suitable for multiplication of the EGMS lines, thus also well suited. Application of the two line breeding system could be more difficult (but not impossible) in the Nile delta in Egypt, where only the August sowing date is predicted to give sterilities less than 50%. In this environment, multiplication of the EGMS line can be issue.

#### Synchronisation of flowering dates of two parents

To produce hybrid seeds, the EGMS line and the male parent must flower in exactly the same week, at a time when the EGMS line is 100% sterile and the local variety is sufficiently fertile. If phenology and sterility parameters of the local popular variety are known we can simulate for both varieties at any flowering date what their sterility will be. Once a desirable flowering date is found, we can trace back from the simulated flowering date the associated sowing dates of the EGMS and the local variety. These can be different due to differences in phenological parameters.

**Fig. 8:**
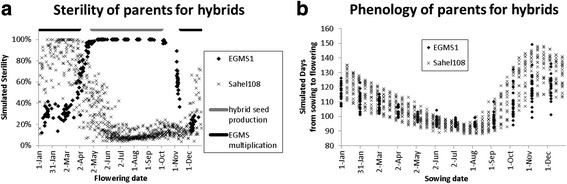
Simulated sterility **a** and days from sowing to flowering **b** of EGMS1 (IR75589-31-27-8-33) and Sahel108. EGMS1 was simulated with sowing on a 31 days timestep Sahel 108 was simulated with sowing on a 10 days timestep. The data points show the variation caused by interannual weather variability, simulations were conducted for the years 1990 to 2015

**Fig. 9:**
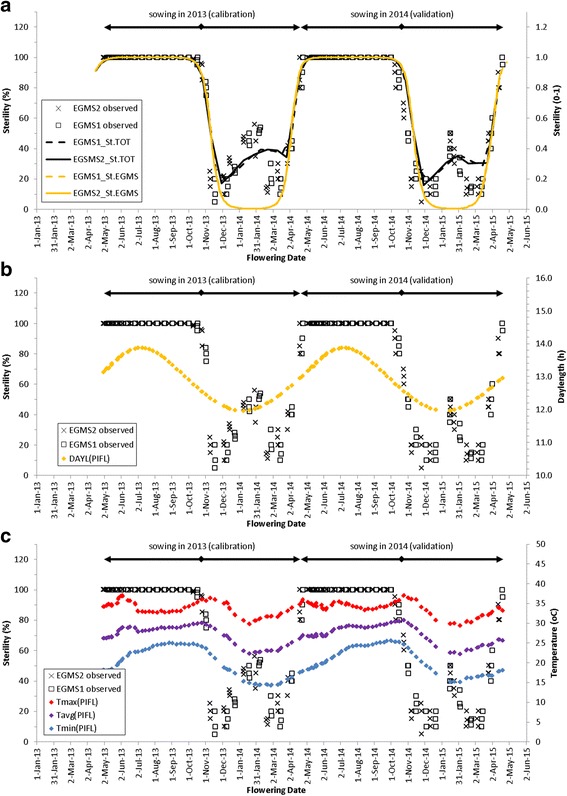
Fertility behaviour of EGMS lines with daylength and temperature. *Black symbols* show observed sterilities for the two EGMS lines. In **a** *black lines* show the simulations with the full model, *yellow lines* show simulated sterility with the EGMS model (without cold sterility). In **b**, the *right* axis and *yellow* symbols show the average daylength in the period from panicle initiation to flowering (PIFL). In **c** the right axis shows air temperature and *coloured symbols* show the average of daily maximum, daily average and daily minimum temperatures, averaged over the PIFL period associated with each flowering date

In Fig. 8 we simulated sterility of the EGMS1 line (this paper) and a local popular variety in the Senegal river valley, Sahel108 (using Sahel108 phenology parameters reported in van Oort et al. 2011 and the model ORYZA2000v2n13s14 reported in van Oort et al. 2015a, with transplanted rice with a seedbed duration of 21 days). We can see in Fig. 8a that when flowering between 1 May and 1 September the EGMS1 line is 100% sterile and the local variety Sahel108 is fertile (<20% sterility). We can also see in this environment it is possible to have multiplication of the EGMS line in one part of the year and production of hybrid seeds in another part of the year. Say that for production of hybrids we want to have both parents flowering on 15 June. Then from phenology simulations (Fig. 8b) we can trace back that Sahel108 must be sown on 20 February and EGMS1 must be sown on 4 March, a difference of 12 days. This example shows how two independent phenology and sterility models can be used to fine tune sowing dates of two parent varieties for production of hybrids. In this case to arrive at the same (synchronised) flowering date, really different sowing dates are required. This shows that understanding of the phenology of the two parents is important because not per definition the two parents must be sown on the same date to have flowering on the same date.

## Discussion

### Practical implications

This paper shows that the two-line hybrid rice breeding system is feasible in the study site (Ndiaye, Senegal) because (1) we found a high (~25%) inheritance of the EGMS trait in the F_2_ population and (2) observations and simulations showed that in this site a long period suitable for producing hybrids (F_1_ seed) through the two line system exists and (3) also a long period suitable for EGMS multiplication exists.

We found a high (~25%) inheritance of the EGMS trait in the F_2_ population. Such is desirable because from this population breeders can select the ~25% sterile plants, or a subset of these 25%, which can then serve as new parents for hybrids. The new parents and the new F_1_ population of hybrids will have, in comparison with the first generation, more “locally desirable” genes. By repeating this process, breeders develop locally adapted hybrids. This whole process would be much less efficient if inheritance were less than 25%, for example 6.25% if two recessive genes were involved. The high (25%) inheritance allows for running a two line breeding system for hybrid rice, which is more efficient than the three line system. In the same time these two EGMS lines can be used directly as male lines with different local variety and breeding lines to produce high yielding F_1_ hybrids.

We presented two possible applications of our model: (1) assessing the suitability of other sites for hybrid rice breeding programs using the same EGMS varieties as considered here and (2) synchronisation of flowering dates and from that selection of suitable sowing dates for the two parent lines for production of hybrids. The suitability analyses identified suitable sowing windows for producing hybrids and for multiplication of the EGMS lines. This can provide useful guidance when establishing new breeding programs in these sites and also provide an opportunity for further validation of the model presented here. The synchronisation exercise showed that the model presented here can be used in combination with other independent existing models for ‘normal’ (=non-EGMS) varieties such as have already been developed and parameterised for many varieties (van Oort et al. 2011, van Oort et al. 2015a, Dingkuhn et al. 2015a,b, Zhang et al. 2016). It should be noted that these two applications serve as a first estimate, which involved applying the model outside the bounds of temperature and daylength for which it was calibrated. We are therefore less certain about the accuracy of these predictions. Validation in these sites is desirable and would be a logical next step if breeding programs are started in these sites. Apart from these two applications particular for rice we observe that the model presented here is very simple requiring estimation of only few parameters. We therefore expect that the same model can be quite easily applied to other EGMS lines in rice and to other crops.

### Scientific implications

The number of genes involved in causing male sterility was not before investigated for the two EGMS lines considered in this paper. A single recessive gene has previously been reported to control male sterility in 5460S (Sun et al. 1989), R59TS (Yang and Wang, 1990), Norin PL 12 (Maruyama et al. 1991), Nongken 58 (Zhuping 1997) and Guangzhan63S (Xu et al. 2011). These lines are more attractive for breeding than those with two or more genes involved, possibly the latter receive less publicity in scientific literature. For the F_2_ of EGMS lines 7001S, 5047S, 5088S previously Zhuping (1997) reported a 1:15 segregation, indicating two recessive genes can also be involved. Those relatively rare cases with two genes involved imply that we cannot take a one-gene controlled EGMS for granted and that it is relevant to check the number of genes involved, as we did in this paper.

Previously, similar models as presented here were developed for simulating phenology (van Oort et al. 2011, Dingkuhn et al. 2015b, Zhang et al. 2016). For photoperiod sensitive rice varieties development can already be delayed at daylengths longer than 10 h (e.g. Yin et al. 1997, Awan et al. 2014). Photoperiod in our study site varied from 12 to 14 h, thus long enough for detecting photoperiod sensitivity if present. Comparisons of model accuracies for phenology simulated with and without photoperiod sensitivity with the pheno_opt_rice2 phenology calibration program (van Oort et al. 2011) showed that for the EGMS lines considered here, accuracy could not be increased by adding photoperiodism. For other EGMS lines, it is relevant to test for photoperiodism and include this in the phenology model if necessary.

Previously, similar models as presented here were developed for heat and cold sterility and applied in the same study area for ‘normal’ varieties, i.e. without male sterility (van Oort et al. 2015a; Julia and Dingkuhn 2013; Dingkuhn et al. 2015a,b). There are two notable differences with the model presented here. Firstly, our EGMS lines have sterility at long days whereas normal varieties do not have this trait. And secondly, cold sterility for our EGMS lines was much smaller than for the varieties studied by van Oort et al. 2015a, Julia and Dingkuhn 2013, Dingkuhn et al. 2015a. For this reason we developed a new sterility model and tested for interaction between daylength and temperature effects. Especially for cold sterility our model was less accurate. Two hypotheses might possibly explain this lower accuracy, but were impossible to test here due to the sparsity of measurements in the cold period and due to lack of more detailed measured phenological data. The first hypothesis is that some process of acclimation is occurring, i.e. plants exposed to cold earlier on in their development might be more tolerant to cold later; this would explain the underestimation of cold sterility of October sown crops (less exposed to early cold, Fig. 2a) and the overestimation of cold sterility of November sown crops (more exposed to early cold, Fig. 2a). The second hypothesis is that we got the period in which rice is sensitive to cold not precisely right and were thus calculating temperature effect over the wrong period. It has been suggested that rice is especially sensitive to cold in a short period around microspore stage. Sensitivity to cold in such a short period is difficult to model, because predictive accuracy becomes strongly dependent on correct estimation of the start and end development stage in which the plant is sensitive.

Also for EGMS sterility it would for further research be interesting to investigate if a certain period within the phase from panicle initiation to flowering is more/less sensitive to daylength. This met with the same problems as noted above: lack of measurements on phenological stages. We used a logistic regression model for modelling the relation between sterility and daylength. The (sigmoid shaped) logisitic model is statistically more appropriate model when analysing binary response variables (sterility) as compared with previous studies that used correlation analysis and linear models (e.g. Latha et al. 2004) or threshold values (e.g. Lopez and Virmani 2000; Virmani et al. 2003). The appropriateness of the logistic model was also clearly shown in the sigmoidal shape of observed sterilities in Fig. 5b.

Cold sterility of the EGMS lines reached a maximum of 50% whereas previously van Oort et al. (2015a), Julia and Dingkuhn (2013) and Dingkuhn et al. (2015a) reported for tropical varieties sterilities of more than 80% when flowering in the same cold December-February period (see also Fig. 7a). The much lower sterility reported there is consistent in that in these previous studies tropical varieties were evaluated, whereas our EGMS lines originated from a cooler environment, thus better adapted to the cold temperatures. Consistently with this, we also found a lower base and optimum temperature for the EGMS lines (*TBD* = 11 °C, *TOD* = 26 °C) than for the tropical varieties normally evaluated for the study site, with *TBD* in the range of 14 to 18 °C and *TOD* in the range of 26 to 34 °C (van Oort et al. 2011). The low sterilities for flowering in the cold December-February period are convenient because they allow for multiplication of EGMS lines during the cold dry season, something which would not be possible with normal varieties (Fig. 8a).

## Conclusions

A newly developed model could accurately simulate phenology and sterility of two EGMS lines grown in Senegal as a function sowing date, weather variables and a limited number of genetic parameters. Daylength from panicle initiation to flowering was the main explanatory variable, additionally also a significant effect of minimum temperatures during the same period was found. The model can be useful for identifying safe sowing windows for production of hybrids and multiplication of the male sterile parent. A statistical analysis of inheritance revealed that only one recessive gene is causing the male sterility. In Senegal, Dry season (February – July) is the suitable period for F_1_ hybrid seed productions and Wet season (August – December) is the suitable period for multiplying the EGMS lines.
